# Supplementary material for: Seminoma and Embryonal Carcinoma Footprints Identified by Analysis of Integrated Genome-Wide Epigenetic and Expression Profiles of Germ Cell Cancer Cell Lines
Source: PLoS One. 2014 Jun 2;9(6):e98330. doi: 10.1371/journal.pone.0098330 (PMC4041891; doi:10.1371/journal.pone.0098330)
Supplement: File S1 — ZIP file containing DMRforPairs output for significant regions. Please start from the html files. (ZIP) [file pone.0098330.s008.zip › figures/12648.pdf]

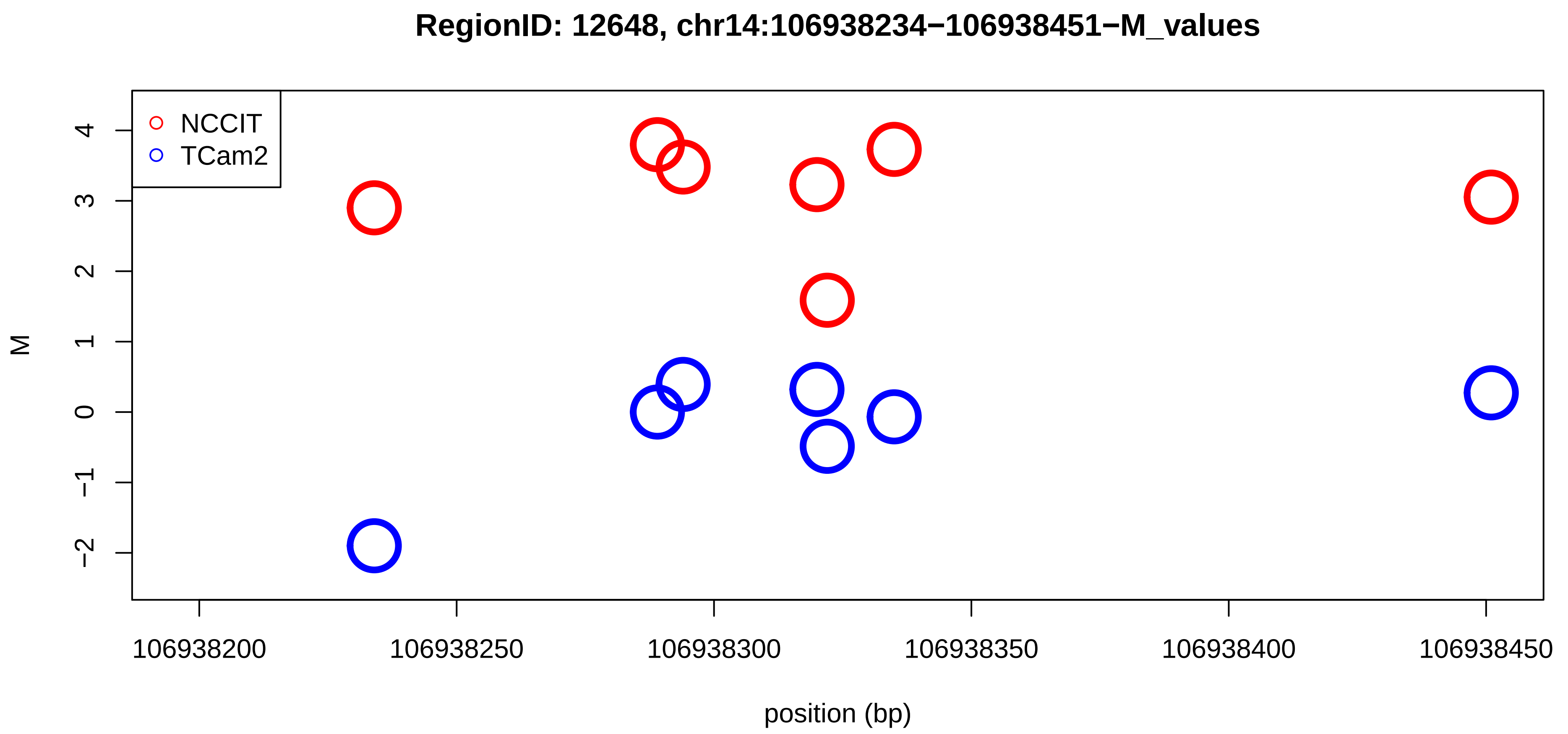
RegionID: 12648, chr14:106938234−106938451−M_values
NCCIT
TCam2
M
−2
−1
0
1
2
3
4
106938200
106938250
106938300
106938350
106938400
106938450
position (bp)

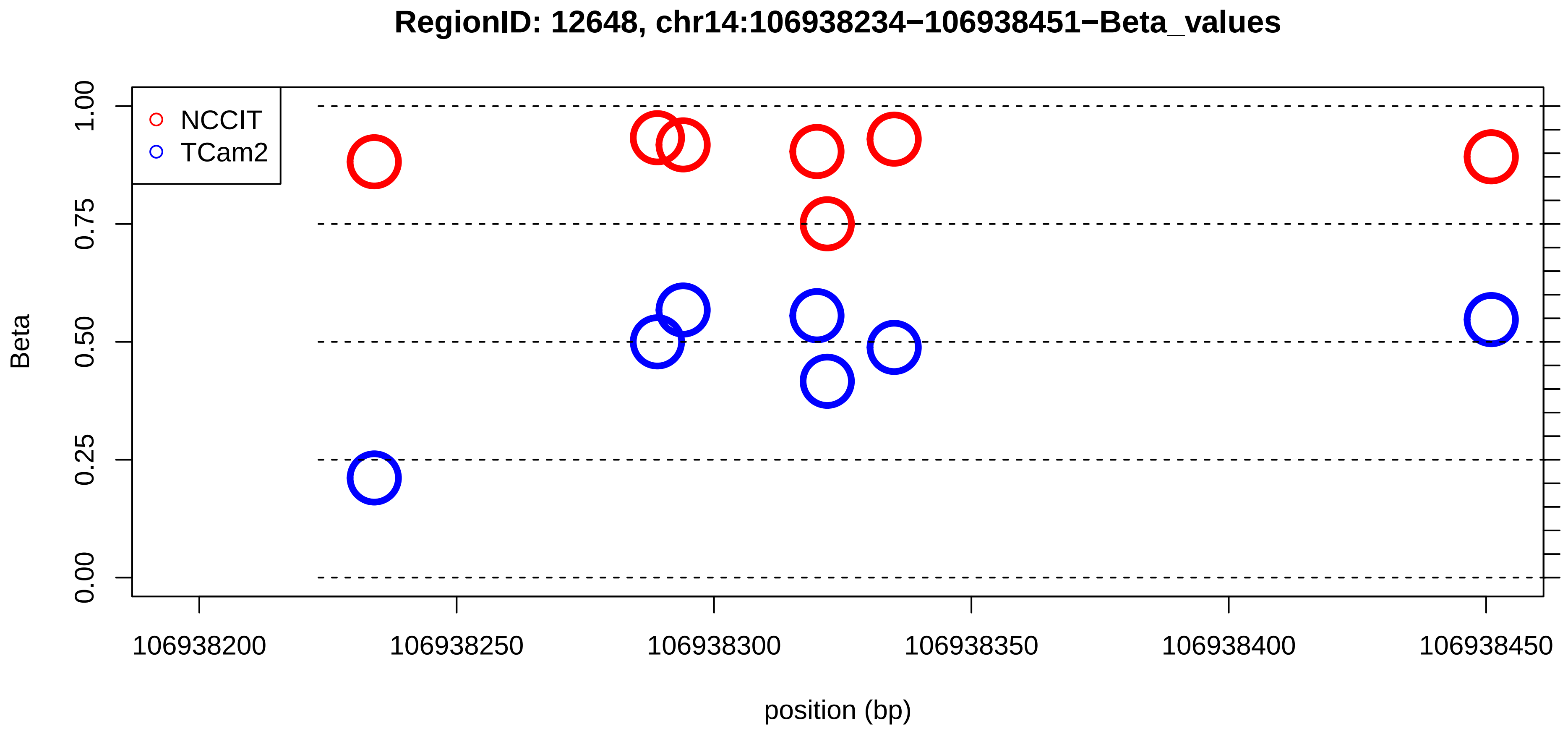
RegionID: 12648, chr14:106938234−106938451−Beta_values
NCCIT
TCam2
Beta
0.00
0.25
0.50
0.75
1.00
106938200
106938250
106938300
106938350
106938400
106938450
position (bp)
